# The current status of tumor microenvironment and cancer stem cells in sorafenib resistance of hepatocellular carcinoma

**DOI:** 10.3389/fonc.2023.1204513

**Published:** 2023-07-27

**Authors:** Siqi Chen, Yaqing Du, Xin-Yuan Guan, Qian Yan

**Affiliations:** ^1^ Guangdong Provincial Key Laboratory of Colorectal and Pelvic Floor Diseases, Guangdong Institute of Gastroenterology, The Sixth Affiliated Hospital, Sun Yat-sen University, Guangzhou, China; ^2^ Department of General Surgery (Colorectal Surgery), The Sixth Affiliated Hospital, Sun Yat-sen University, Guangzhou, China; ^3^ Biomedical Innovation Center, The Sixth Affiliated Hospital, Sun Yat-sen University, Guangzhou, China; ^4^ Institute of Basic Medical Sciences, School of Life Sciences and Biopharmaceuticals, Guangdong Pharmaceutical University, Guangzhou, China; ^5^ State Key Laboratory for Liver Research, The University of Hong Kong, Hong Kong, Hong Kong SAR, China

**Keywords:** hepatocellular carcinoma, sorafenib resistance, tumor microenvironment, cancer stem cells, hypoxia, immune microenvironment, EMT, exosomes

## Abstract

Hepatocellular carcinoma (HCC) is a heterogeneous and aggressive liver cancer that presents limited treatment options. Despite being the standard therapy for advanced HCC, sorafenib frequently encounters resistance, emphasizing the need to uncover the underlying mechanisms and develop effective treatments. This comprehensive review highlights the crucial interplay between the tumor microenvironment, cancer stem cells (CSCs), and epithelial-mesenchymal transition (EMT) in the context of sorafenib resistance. The tumor microenvironment, encompassing hypoxia, immune cells, stromal cells, and exosomes, exerts a significant impact on HCC progression and therapy response. Hypoxic conditions and immune cell infiltration create an immunosuppressive milieu, shielding tumor cells from immune surveillance and hindering therapeutic efficacy. Additionally, the presence of CSCs emerges as a prominent contributor to sorafenib resistance, with CD133+ CSCs implicated in drug resistance and tumor initiation. Moreover, CSCs undergo EMT, a process intimately linked to tumor progression, CSC activation, and further promotion of sorafenib resistance, metastasis, and tumor-initiating capacity. Elucidating the correlation between the tumor microenvironment, CSCs, and sorafenib resistance holds paramount importance in the quest to develop reliable biomarkers capable of predicting therapeutic response. Novel therapeutic strategies must consider the influence of the tumor microenvironment and CSC activation to effectively overcome sorafenib resistance in HCC.

## Introduction

Primary liver cancer was identified as the sixth most commonly diagnosed cancer and the third leading cause of cancer-related deaths worldwide in 2020, ranking only below lung and colorectal cancer in terms of mortality rates (1). The predominant form of primary liver cancer is hepatocellular carcinoma (HCC), accounting for 75% to 85% of all cases (1). Despite significant advancements in HCC treatment, clinical outcomes remain unsatisfactory, with over half of patients experiencing relapse within five years (2).

For patients with advanced-stage HCC, the first-line treatment since 2008 has been the multi-kinase inhibitor sorafenib, which has demonstrated efficacy in extending patients’ survival time (3). However, the development of drug resistance typically occurs within six months of treatment (4), necessitating the urgent exploration of novel therapeutic regimens to overcome sorafenib resistance.

Sorafenib, a multi-target tyrosine kinase inhibitor (TKI), has exhibited potent anti-angiogenic and anti-proliferative effects on tumor cells. Notably, sorafenib achieves tumor angiogenesis inhibition by targeting the hepatocyte cytokine receptor, platelet-derived growth factor receptor, and vascular endothelial growth factor receptor 2 (VEGFR-2) (5). Furthermore, it hinders tumor proliferation by focusing on RAF-1, b-Raf, and kinase activity in the Ras/Raf/MEK/ERK signaling pathway (6). Despite the introduction of alternative treatment strategies such as lenvatinib, regorafenib, and immune checkpoint inhibitors, sorafenib retains a prominent position in clinical settings, supported by substantial evidence.

**Figure 1:**
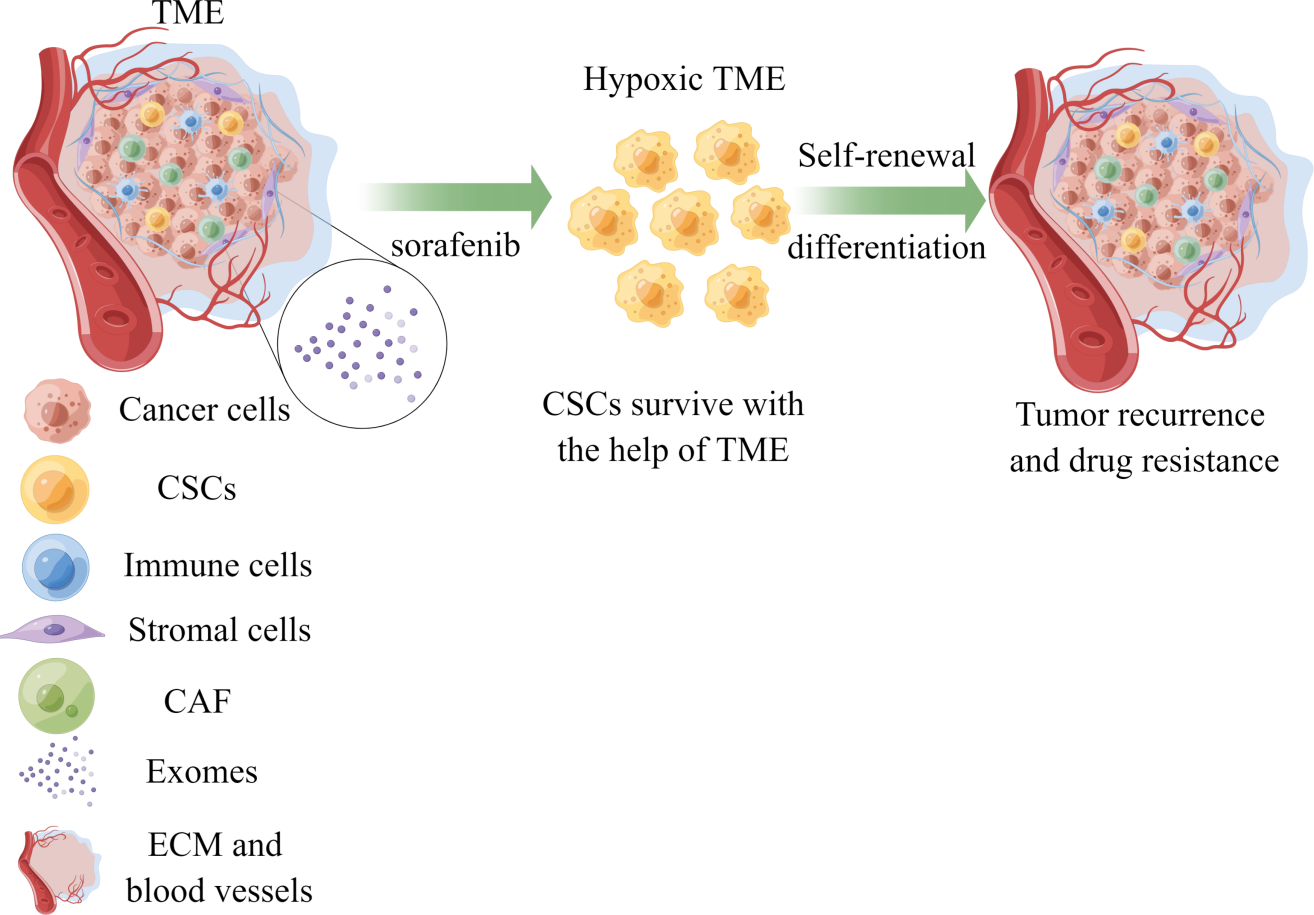
The role of TME and CSCs in sorafenib resistance of HCC. Cancer stem cells could survive with the help of components of TME under the treatment of sorafenib, leading to drug resistance and tumor recurrence. TME, tumor microenvironment; CSCs, cancer stem cells; CAF, cancer-associated fibroblasts; ECM, extracellular matrix; HCC, hepatocellular carcinoma.

Elucidating the factors contributing to sorafenib resistance holds immense significance in improving treatment outcomes for hepatocellular carcinoma (HCC) patients. Recent studies have emphasized the crucial roles played by the tumor microenvironment (TME) and cancer stem cells (CSCs) in drug resistance development. In this review, we provide an overview of recent findings regarding hypoxia, the immune microenvironment, viral reactivation, and exosomes within the TME, alongside the distinctive features of CSCs that contribute to sorafenib resistance. This comprehensive understanding facilitates the advancement of novel clinical implementations and treatment selection (**Figure 1**).

## Tumor microenvironment and sorafenib resistance of HCC

The tumor microenvironment (TME) comprises tumor cells, cancer-associated stromal cells, immune cells, secreted products (such as cytokines and chemokines) from these cells, and non-cellular components of the extracellular matrix (ECM) (7). The TME significantly influences tumor initiation and progression. Recent studies have underscored the critical role of interactions between tumor and non-tumor cells in hepatocellular carcinoma (HCC) metastasis (3). Furthermore, non-cellular proteins or molecules within the TME have been implicated in profoundly affecting tumor cell behavior (2). The major effects and molecular signaling of TME components on sorafenib resistance in HCC are summarized in **Table 1**.

**Table 1:** The role of TME in sorafenib resistance of HCC.

Research area	Molecules/Drugs/cells	Major effects	Pathway	Resources	References
Hypoxia	HIF-1α	the reduction of VEGF expression and tumor angiogenesis in HCC	HIF-1α/VEGF	*in-vitro*	(8, 9)
	NF-κB	EF24 overrides sorafenib resistance through VHL-dependent HIF-1α degradation and NF-κB inactivation.	–	*in-vitro* and *in-vivo*	(10)
	HIF-1α and HIF-2α	indispensable for HCC cells developing sorafenib resistance and re-acquiring angiogenesis and proliferation	–	*in-vitro* and *in-vivo*	(11, 12)
	Sorafenib	activating the expression of VEGF and cyclin D1	HIF-1α- to HIF-2α-dependent pathways	observational studies in patients	(13, 14)
		increased HIF-2α expression induced by sorafenib leads to drug resistance	TGF-α/EGFR	*in-vitro*	(15)
	Mir-338-3p	sensitized the HCC cells to sorafenib by targeting HIF-1α	–	*in-vitro* and *in-vivo*	(16)
	HIF-2α inhibitor PT-2385	improve sorafenib efficacy	the androgen receptor and the pSTAT3/pAKT/pERK	*in-vitro* and *in-vivo*	(17)
	PFH@LSLP	improve sorafenib efficacy	CSF1/CSF1R	*in-vivo*	(18)
Immune microenvironment	TAMs	induce EMT and enhance stemness features	–	*in-vitro* and observational studies in patients	(19, 20)
	M2 macrophages	promote tumor growth, migration, and invasion	–	*in-vitro*	(21)
	HGF	the drug resistance of HCC	HGF/C-Met, ERK1/2/MAPK and PI3K/AKT		
	the neutrophils	might promote or suppress cancer cells proliferation and metastasis	–	*in-vitro* and observational studies in patients	(22)
	Sorafenib	Increase the infiltration level of TANs	–		
		Tumor immunosuppression caused by hypoxia was also observed with continues sorafenib treatment	–	*in-vivo*	(23)
	CCR4^+^ Treg	can enhance antitumor immunity, overcome sorafenib resistance, and sensitize tumors to PD-1 checkpoint blockade.	–	*In-vivo* and *in-vitro*	(24)
Exosomes	Mir-122	significantly improved the antitumor effect of sorafenib in HCC mice models	–	*in-vitro*	(25)
	Si-GRP78-modifed exosomeslncRNA-VLDLR	incubation of tumor cells with EVs containing lncRNA-VLDLR significantly reduced their sensitivity to sorafenib	–	*in-vitro*	(26)
		inhibit HCC cells proliferation and invasion, as well as elevate the sensitivity to sorafenib treatment by targeting the oncogene GRP78	–	*in-vitro* and *in-vivo*	(27)

### Hypoxia

The hypoxic environment in hepatocellular carcinoma (HCC) contributes to angiogenesis, invasiveness, and chemoresistance by upregulating the expression of vascular endothelial growth factor (VEGF) and hypoxia-inducible factor 1α (HIF-1α) (8). HIFs are transcription factors (TFs) composed of key members such as HIF-1α, HIF-2α, HIF-3α, and HIF-β, which regulate tumor proliferation, angiogenesis, and metastasis through hypoxia-induced signaling pathways. Previous studies have demonstrated the crucial roles of both HIF-1α and HIF-2α in HCC initiation (9). Overexpression of HIF-1α has been observed in HCC patients and correlates with poor clinical outcomes (9).

A causal loop exists between acquired sorafenib resistance and the hypoxic tumor microenvironment. Multiple studies have indicated that continuous sorafenib treatment induces tumor hypoxia, leading to the selection of tumor clones that adapt to a hypoxic environment by activating HIF-1α and NF-κB, thereby diminishing the efficacy of sorafenib (10). Nuclear translocation of HIF-1α and HIF-2α is indispensable for HCC cells to develop sorafenib resistance and regain angiogenesis and proliferation capabilities (11, 12). Other studies have found that sorafenib triggers a switch from HIF-1α-dependent to HIF-2α-dependent pathways, promoting the expression of VEGF and cyclin D1, thus enhancing sorafenib resistance and tumor growth (13, 14). Increased expression of HIF-2α induced by sorafenib activates the TGF-α/EGFR pathway, leading to drug resistance (15). Given that HIF proteins play a significant role in sorafenib resistance, various approaches have been explored to target HIF proteins using small molecules or microRNAs (28). For example, Xu et al. reported that Mir-338-3p sensitized HCC cells to sorafenib by targeting HIF-1α (16). Additionally, the HIF-2α inhibitor PT-2385 was found to significantly improve sorafenib efficacy by upregulating the androgen receptor and suppressing downstream pSTAT3/pAKT/pERK signaling (17). Collectively, these findings highlight the tight association between abnormal expression of HIF proteins and sorafenib resistance in HCC, suggesting a causal relationship between hypoxia and the therapeutic efficacy of sorafenib. On the other hand, clinical sorafenib treatment can activate the C-X-C receptor type 4 (CXCR4)/stromal-derived factor-1α (SDF-1α) axis, aggravate intratumoral hypoxia in hepatocellular carcinoma (HCC), and further lead to hepatocellular carcinoma. Therefore, a multifunctional oxygen delivery nanoplatform was rationally constructed based on oxygen-saturated perfluorohexane (PFH) core liposomes, with the CXCR4 antagonist LFC131 peptide modified on the surface to simultaneously deliver sorafenib and the CSF1/CSF1R inhibitor PLX3397 (named PFH@LSLP) for the treatment of HCC resistant to sorafenib (18). Targeting these molecules could offer an effective therapeutic strategy to overcome sorafenib resistance in HCC.

### Immune microenvironment

The immune microenvironment plays a crucial role in the malignant transformation and progression of hepatocellular carcinoma (HCC), and significant advancements have been made in immunotherapy strategies in recent years. Emerging evidence suggests a close correlation between the tumor immune microenvironment and drug resistance. For instance, the infiltration of tumor-associated macrophages (TAMs) has been reported to be involved in regulating the sensitivity of HCC to sorafenib (29–31). Studies have shown that TAM infiltration occurs concomitantly with the tumor epithelial-to-mesenchymal transition (EMT) status (19). This phenomenon may be attributed to the ability of TAMs to induce EMT and enhance stemness features in tumor cells, contributing to sorafenib resistance (20). However, the underlying mechanisms of TAM-mediated tumor cell progression remain largely unknown. Cytokines or chemokines secreted by TAMs are often the key mediators of communication between TAMs and cancer cells. For instance, hepatocyte growth factor (HGF) produced by M2 macrophages has been shown to promote tumor growth, migration, and invasion (21). Infiltration levels of M2 TAMs and HGF expression were found to be significantly increased in drug-resistant tumor cells compared to sorafenib-sensitive tumors (21). Mechanistic studies have revealed that HGF mediates drug resistance in HCC through the activation of the HGF/C-Met, ERK1/2/MAPK, and PI3K/AKT pathways (21). Considering the pivotal role of TAMs in supporting cancer cell survival and progression, therapeutic agents targeting TAMs are being rapidly developed, with some clinical trials demonstrating promising results and improved clinical outcomes (32).

Neutrophils, another component of the immune microenvironment, exert significant influence on the behavior of cancer cells. The impact of neutrophils on cancer cell proliferation and metastasis can vary depending on the specific tumor microenvironment (31). Zhou and colleagues investigated the role of tumor-associated neutrophils (TANs) in HCC progression and found that patients who received preoperative sorafenib treatment exhibited significantly higher levels of TAN infiltration compared to those who did not receive sorafenib. This finding was further validated in xenograft mouse models, where sorafenib treatment led to increased TAN infiltration along with enhanced expression of CCL2 and CCL17. Depletion of TANs combined with sorafenib administration achieved the greatest suppression of tumor growth in mice compared to using sorafenib alone (31).

Over more, cancer-associated fibroblasts (CAFs) play a significant role in chemo-resistance induction, promoting tumor cell proliferation, invasiveness, and metastatic potential in various cancers, including HCC (33, 34). CAF-derived SPP1 was identified to enhance TKI resistance in HCC by bypassing oncogenic signaling activation and promoting EMT. Targeting CAF-derived SPP1 represents a promising therapeutic approach against TKI resistance in HCC (35).

Furthermore, the immune microenvironment is regulated by hypoxia. Studies have demonstrated that sorafenib-induced hypoxia can upregulate the expression of CXCL5 and IL-1β in HCC cells in a HIF-dependent manner (22, 31). This, in turn, recruits peripheral blood neutrophils to the tumor site, where they differentiate into TANs. Under hypoxic conditions, TAMs and regulatory T cells (Tregs) are also activated, collectively promoting tumor angiogenesis (31). Gao et al. revealed that targeting intratumoral stem-like CCR4+ regulatory T cells (Tregs) enhances antitumor immunity, overcomes sorafenib resistance, and sensitizes tumors to PD-1 checkpoint blockade. Inhibiting Treg infiltration into the tumor microenvironment using a CCR4 antagonist or N-CCR4-Fc effectively reduces Treg accumulation. These findings suggest that targeting intratumoral CCR4+ Tregs holds promise as a therapeutic strategy to improve immune responses and overcome sorafenib resistance (24). Continued sorafenib treatment has been observed to cause tumor immunosuppression characterized by increased expression of programmed death ligand 1 (PD-L1) and accumulation of Treg cells, leading to tumor recurrence (36, 37). In light of these observations, the combination of immune checkpoint blockade with sorafenib holds great promise for the treatment of HCC.

### Exosomes

Exosomes, which are small extracellular vesicles (EVs) derived from stromal cells or tumor cells, play a critical role in cell-to-cell communication within the tumor microenvironment (TME). Numerous studies have highlighted the involvement of exosomes in the regulation of sorafenib sensitivity both *in vitro* and *in vivo* (23). For instance, exosomes secreted by adipose tissue mesenchymal stem cells (AMSCs) were found to carry and deliver Mir-122, a microRNA that mediates the communication between AMSCs and HCC cells. Mir-122, upon transport into tumor cells, regulates the sensitivity of HCC to sorafenib by modulating the expression of target genes. Intratumoral injection of Mir-122-loaded exosomes significantly enhanced the anti-tumor effect of sorafenib in HCC mouse models, underscoring the therapeutic potential of exosomes in cancer treatment (25). In addition to microRNAs, long non-coding RNAs (lncRNAs) have also been implicated in exosome-mediated drug resistance. Studies have revealed that sorafenib treatment increases the expression of lncRNA-VLDLR in tumor cells, which subsequently leads to the release of EVs. Incubation of tumor cells with EVs containing lncRNA-VLDLR significantly reduces their sensitivity to sorafenib, confirming the involvement of exosomes in drug resistance (26).

Given the involvement of exosomes in various aspects of tumor behavior, exosomes loaded with specific functional molecules hold promise as an effective therapeutic strategy. For instance, Li et al. demonstrated that exosomes modified with Si-GRP78, which targets the oncogene GRP78, inhibit HCC cell proliferation and invasion while enhancing sensitivity to sorafenib treatment (27). These findings collectively suggest that exosome-mediated intercellular communication can be harnessed to develop innovative strategies for overcoming drug resistance in cancer treatment.

## Cancer stem cell and sorafenib resistance of HCC

Cancer stem cells (CSCs) represent a distinct subset of tumor cells that possess stem cell-like properties, including self-renewal and the ability to differentiate (38). The presence of CSCs contributes to tumor heterogeneity, therapy resistance, and tumor recurrence (39). The origin of CSCs is a subject of ongoing debate. Some studies suggest that CSCs may arise from tissue stem cells, while others propose that they originate from dedifferentiation or cell fate transition of tumor cells (38). Emerging evidence indicates that the epithelial-mesenchymal transition (EMT) process plays a role in the generation of CSCs associated with drug resistance (39). In this review, we focus on the potential mechanisms underlying sorafenib resistance mediated by CSCs, taking into account the characteristics of EMT and tumor heterogeneity. Furthermore, we discuss how this knowledge may contribute to the development of novel therapeutic approaches to overcome sorafenib resistance in advanced HCC.

### CSCs contribute to tumor heterogenicity

Hepatocellular carcinoma (HCC) exhibits high genomic and transcriptomic heterogeneity, contributing to its ability for unlimited proliferation, distant metastasis, and therapy resistance (40). This heterogeneity is driven by the self-renewal and differentiation capabilities of cancer stem cells (CSCs), which give rise to distinct subpopulations within the tumor with varying sensitivity to treatment (41). Single-cell RNA sequencing studies have revealed that CSC populations differ among HCC patients, underscoring the heterogeneity of CSCs (42, 43). Key transcription factors such as NANOG, SOX2, OCT4, KLF4, and c-MYC regulate the stemness features of CSCs, enabling tumors to sustain self-renewal (44). Various surface markers, including CD133, CD90, CD24, and CD44, are utilized to identify and isolate CSCs from the bulk tumor. CD133+ CSCs have been associated with increased drug resistance and tumor initiation potential (44).

Developmental signaling pathways, such as Hedgehog, Hippo, Notch, TGF-β, and Wnt/β-catenin, play crucial roles in regulating CSCs and driving tumor heterogeneity, therapy resistance, and cancer progression (45). For instance, Notch signaling components, including Notch and Jagged proteins, are significantly upregulated in CD133+ CSCs of HCC (46). Researchers have identified an expanding repertoire of biomarkers and driver genes involved in the activation of CSCs. Targeting these drivers may hold promise for eliminating CSCs and reversing therapeutic resistance in HCC. Recently, a research find the overexpression of SERPINA12 in hepatocellular carcinoma (HCC) is associated with aggressive clinical features and stemness characteristics. This upregulation is mediated by TCF7L2 binding to the SERPINA12 promoter, resulting in enhanced transcriptional activity (47). SERPINA12 interacts with GRP78, activating the AKT/GSK3β/β-catenin pathway and forming a positive feedback loop. In an immunocompetent HCC mouse model, intravenous administration of rAAV8-shSERPINA12 sensitized HCC cells to sorafenib and suppressed the cancer stem cell subset. Targeting SERPINA12 holds promise for improving sorafenib efficacy and inhibiting HCC stemness (47). Meanwhile, Luo et al. found that GLI1 editing promotes metabolic shift to oxidative phosphorylation (OXPHOS) to control stress and stem cell-like state through PINK1-Parkin-mediated mitophagy in HCC, thereby endowing it with unique metastatic and sola Fenil resistance (48).

### CSCs undergo EMT

Indeed, the epithelial-mesenchymal transition (EMT) process plays a critical role in the regulation of cancer stem cell (CSC) features and contributes to tumor aggressiveness and therapy resistance. EMT is a complex cellular reprogramming process in which epithelial cells lose their differentiated characteristics and acquire mesenchymal traits (49). Tumor cells undergoing EMT exhibit enhanced migratory, invasive, and anti-apoptotic capabilities (50). Growing evidence suggests that EMT is closely intertwined with CSCs and influences their behavior (51).

During EMT, tumor cells can undergo a reversible transition to a mesenchymal phenotype, leading to the acquisition of CSC-like properties, such as increased metastatic potential and tumor-initiating capacity. For example, liver CSCs characterized by the expression of the RALY RNA binding protein like (RALYL) exhibit reversible EMT states and demonstrate enhanced metastasis and tumor initiation abilities (52). Poorly differentiated HCC with stem cell characteristics is strongly associated with an EMT phenotype, which is often associated with a lower response to chemotherapy and poorer survival outcomes (39).

Several studies have demonstrated that CSC enrichment and activation of EMT signaling pathways are associated with sorafenib resistance in HCC (53–55). Furthermore, the induction of EMT and CSC signaling has been identified as key mechanisms contributing to tumor recurrence in HCC patients treated with radiofrequency ablation (RFA) for local control of the disease (56, 57). Moreover, the researchers shed light on the presence of a small subset of quiescent stem-like cells within HCC cell populations, which exhibit intrinsic resistance to sorafenib. Through comprehensive investigations utilizing single-cell sequencing and *in vitro* experiments, the study provides compelling evidence elucidating the pivotal role of stemness and EMT in driving sorafenib resistance at the single-cell level. The findings underscore the importance of considering the unique characteristics of these quiescent stem-like cells and their association with EMT in the development of targeted therapeutic strategies to overcome sorafenib resistance in HCC (58). Understanding the intricate relationship between CSCs and EMT is crucial for the development of novel treatment approaches to overcome sorafenib resistance in HCC. By targeting the molecular pathways involved in CSCs and EMT, researchers may uncover potential therapeutic strategies to improve the outcomes of HCC patients and reduce tumor recurrence.

## Conclusions

Indeed, the tumor microenvironment, including factors such as hypoxia, immune cells, stromal cells, and exosomes, plays a crucial role in promoting sorafenib resistance in HCC. Additionally, CSCs and EMT activation contribute to drug resistance, tumor metastasis, and relapse. In conclusion, future studies should focus on elucidating the correlation between the tumor microenvironment, cancer stemness, and sorafenib sensitivity, as well as identifying reliable biomarkers for predicting the response to sorafenib. Additionally, novel therapeutic methods targeting sorafenib-resistant HCC should be developed, considering the influence of the tumor microenvironment and CSC activation. By addressing these challenges, we can advance our understanding and develop effective strategies to overcome sorafenib resistance in HCC.

## Author contributions

SQC wrote a paper, including manuscripts, images, and tables. QY and XYG provided guidance and suggestions for the paper, and made revisions to the paper. YQD helped modify part of the content.
